# Post-PRRT scans: which scans to make and what to look for

**DOI:** 10.1186/s40644-022-00467-1

**Published:** 2022-06-17

**Authors:** Else A. Aalbersberg, Daphne M. V. de Vries–Huizing, Margot E. T. Tesselaar, Marcel P. M. Stokkel, Michelle W. J. Versleijen

**Affiliations:** 1 Department of Nuclear Medicine, Plesmanlaan 121, 1066 CX Amsterdam, the Netherlands; 2 Department of Medical Oncology, Antoni van Leeuwenhoek Hospital, Amsterdam, the Netherlands

**Keywords:** PRRT, Post-therapy imaging, Planar imaging, SPECT/CT, [^177^Lu]Lu-HA-DOTATATE, Ascites

## Abstract

**Aim:**

The aim of this study was to evaluate the clinical utility of SPECT/CT (imaging of uptake in tumor lesions and additional findings) and the additional value of planar imaging in order to simplify clinical imaging protocols and decrease patients burden.

**Materials and methods:**

One hundred consecutive patients with metastatic neuroendocrine tumor (NET) treated with PRRT were included. Post-therapy imaging was performed 24 h after each PRRT cycle by both whole-body planar imaging and abdominal- and thoracic SPECT/CT. All images were evaluated for (1) the presence of new lesions, (2) discordant lesions between the two acquisitions (planar or SPECT), (3) location of lesions on SPECT (abdominal, thoracic, or both), and (4) additional findings on non-contrast enhanced CT imaging.

**Results:**

In total 368 PRRT cycles including post-therapy imaging were performed in 100 patients. 45 patients had abdominal disease only, whilst in 55 patients the disease was observed on both abdominal and thoracic SPECT. 16 patients had known bone lesions that were visible only on planar imaging as these were out of range of the SPECT/CT. During PRRT, one patient developed multiple new bone metastases after the second cycle of PRRT, which were visible on both planar and SPECT/CT images. In 11 patients additional findings were found on CT images, the most common and relevant being bowel obstruction, pleural effusion, and ascites. Patients who developed ascites during PRRT appeared to do extremely poor; a post-hoc analysis showed that overall survival was 13.2 months in patients that showed ascites during PRRT at any moment and 37.9 months in patients without ascites (*p* < 0.001).

**Conclusion:**

From a clinical point of view, thoracoabdominal SPECT/CT imaging is the preferred method for post-PRRT imaging; planar imaging had no added value over SPECT/CT in this cohort. In patients with abdominal disease only on baseline imaging, SPECT/CT of the abdomen only might be sufficient for imaging during the PRRT course. All accompanying CT images should be reviewed for additional findings, especially ascites, which is suggested to be a poor prognostic factor in patients receiving PRRT.

## Introduction

Neuroendocrine tumors (NETs) are a rare entity, most often found in the gastroenteropancreatic tract or lung. A common characteristic in this heterogeneous group of tumors is the overexpression of the somatostatin receptor (SSTR) [1], which is exploited for both diagnostic and therapeutic purposes. Peptide receptor radionuclide therapy (PRRT) consists of somatostatin analogues (SSAs) coupled to the beta-emitting isotope Lutetium-177 (^177^Lu) and has become an established treatment for advanced NETs [2]. PRRT usually comprises 4 cycles of 7.4 GBq [^177^Lu]Lu-DOTATATE administered at 6–12 week intervals [3]. In case of toxicity, the administered activity can be reduced or the time interval between cycles extended [4].

In addition to beta-radiation for therapeutic effects, ^177^Lu also emits gamma photons with two energy peaks at 113 keV and 208 keV, enabling post-therapy gamma imaging of [^177^Lu]Lu-DOTATATE [5]. Many different protocols exist that vary in timing, acquisition protocol, number of scans, and type of scans (planar and/or SPECT) generated. Scientific publications and guidelines concerning post-therapy imaging mainly focus on dosimetry to calculate tumor- and/or normal tissue dose to personalize therapy [6]. In addition, far less frequent reasons for post-therapy imaging are either quality assurance (successful administration of therapy) or early identification of disease progression during therapy. Observations from post-therapy imaging, for example detection of new lesions and uptake in known lesions, are rarely described in current literature. One study examined rapid (10 min) quality assurance whole-body planar imaging in 16 patients concluding that this can be used for a preliminary assessment of disease course during treatment [7]. The aim of the current study was to evaluate the utility of SPECT/CT (imaging of uptake in tumor lesions and additional findings) and the additional value of planar imaging in order to simplify clinical imaging protocols, reduce scanning time, and thereby decrease patient burden.

## Materials and methods

### Patient selection and treatment protocol

This retrospective study includes 100 consecutive patients with confirmed advanced NET treated with PRRT between March 2016 and June 2019 and was approved by the local Institutional Research Board. Pre-therapy imaging consisted of [^68^ Ga]Ga-HA-DOTATATE PET/CT within 6 months prior to start of therapy, [^18^F]FDG PET/CT, renography with [^99m^Tc]Tc-MAG3 and contrast-enhanced CT scan of (thorax and) abdomen within 2 months prior to start of therapy. In addition, hematological assessment was performed within 1 month prior to start of therapy. In order to be considered a good candidate for PRRT, tumor lesions (including those positive for [^18^F]FDG) should show sufficient uptake of [^68^ Ga]Ga-HA-DOTATATE (higher than normal liver uptake), the renal outflow should be unobstructed and hematological values should be as follows: hemoglobin > 5.5 mmol/L, leucocyte counts > 3.0 × 10^9^/L, neutrophil granulocyte counts > 1.0 × 10^9^/L, platelet counts > 75 × 10^9^/L, kidney function measured by the glomerular filtration rate > 50 ml/min/1.7m2, bilirubin maximal three times upper limit of normal and serum albumin > 30 g/L. If all criteria were met, patients were treated with four cycles of 7.4 GBq [^177^Lu]Lu-HA-DOTATATE with ten-weeks intervals combined with amino acid infusion for renal protection.

### Post-therapy scans: acquisition

All patients received post-therapy imaging 24 ± 3 h after each [^177^Lu]Lu-HA-DOTATATE administration, acquired on Symbia T systems (Siemens Healthineers, Erlangen, Germany) equipped with a medium energy low penetration collimator. First, whole-body (top of skull to toe) anterior and posterior planar images were acquired at a speed of 15 cm/min using the primary photopeak at 208 keV ± 10%. Subsequently, a SPECT/CT of the thorax and abdomen was acquired, with the primary energy window positioned at 208 keV ± 10% with one downscatter (166.4–187.2 keV) and two general scatter windows (56.1–166.0 keV and 18.5–55.5 keV). The SPECT parameters consist of a matrix size of 128*128 with 3.7 × 3.7 × 5.0 mm voxels, non-circular continuous rotation of 180° per head, 96 views per head, and 13 s per view. SPECT reconstruction included attenuation and scatter corrected 3DOSEM (FLASH3D) with 4 iterations and 8 subsets without post-reconstruction filtering. A low-dose non-contrast-enhanced CT scan was made with 40 mAs and 130 kV. In practice, whole-body planar imaging takes approximately 15–20 min, a single-bed SPECT/CT 20 min, and a double-bed SPECT/CT 40 min.

### Post-therapy scans: clinical evaluation

The clinical value of the different post-therapy [^177^Lu]Lu-HA-DOTATATE gamma acquisitions was investigated using four criteria. First, the presence of new lesions was evaluated, defined as lesions that appear on post-therapy imaging that had not been previously identified on the pre-therapy [^68^ Ga]Ga-HA-DOTATATE PET/CT. Second, the presence of lesions detected solely on planar imaging, due to the limited field-of-view of the SPECT/CT, was assessed. Third, the value of both thoracic and abdominal SPECT/CT was determined. For this purpose, patients were divided into three groups based on the localization of their disease on pre-therapy [^68^ Ga]Ga-HA-DOTATATE PET/CT: thoracic disease only, abdominal disease only, and both thoracic and abdominal disease. Finally, all SPECT/CT studies were reviewed for additional findings on the low-dose CT. Imaging analysis was performed using OsiriX Medical Viewer (Pixmeo Sarl, v.10.0.4).

## Results

### Patient characteristics

One-hundred patients were included; 48 male and with a mean age of 63 years at start of therapy. Forty-five patients had a grade 1 NET, 49 grade 2, 5 grade 3, and 1 unknown (grading not performed). The primary tumor was most often located in the small intestine (*n* = 51), followed by the pancreas (*n* = 27), other location in the intestine (*n* = 7), lung (*n* = 5) or unknown (*n* = 10). Eighty-two patients received four PRRT cycles, seven received three cycles, eight received two cycles, and three patients received one cycle of PRRT, resulting in 368 post-therapy scans. Therapy was discontinued due to bone marrow toxicity in 6 cases, disease progression in 5 cases, or clinical deterioration in 7 cases.

### Identification of new lesions

**Fig. 1:**
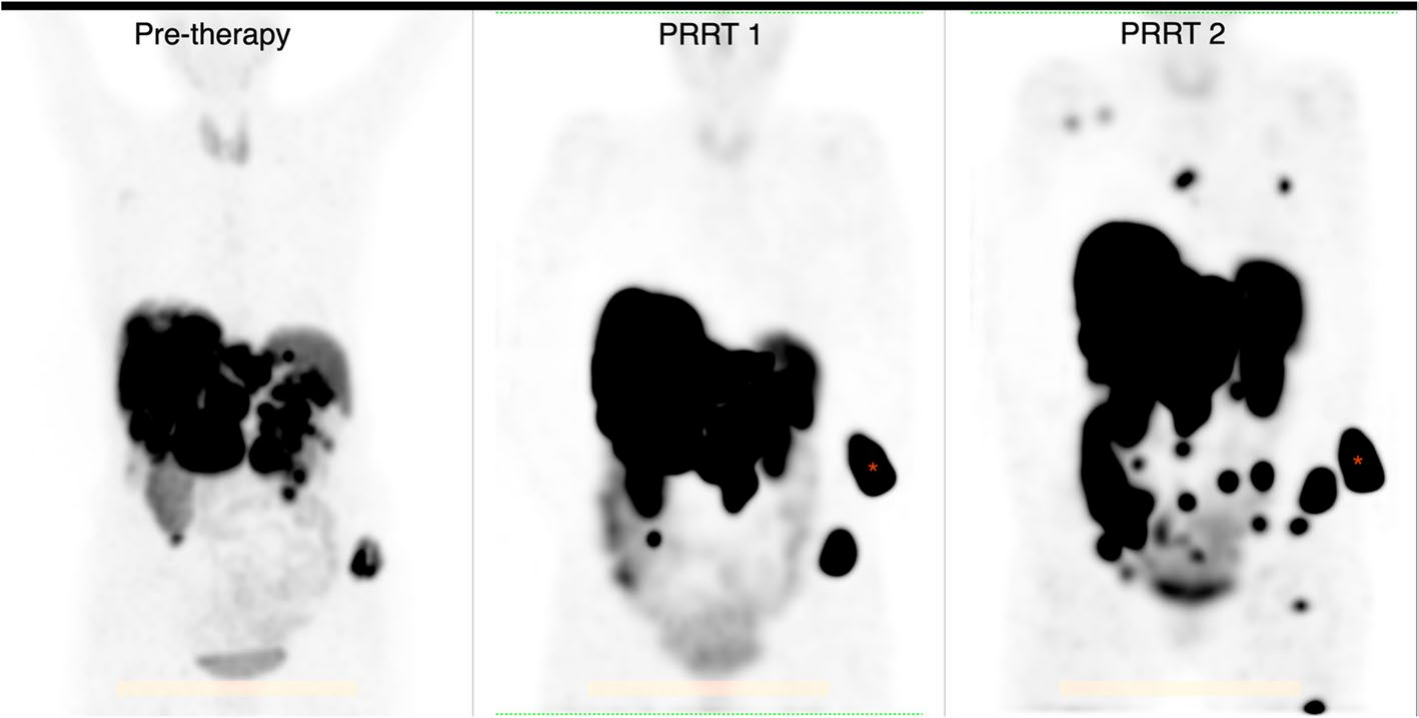
Patient that developed new lesions during PRRT. Maximum intensity projections of the pre-therapy [^68^ Ga]Ga-HA-DOTATATE PET/CT (left), SPECT/CT after PRRT cycle 1 (middle), and SPECT/CT after cycle 2 (right) are shown. Multiple new bone lesions are seen after cycle 2. * indicates standard for quantification (not tumor)

Three patients (3%) presented with lesions on the post-therapy imaging that were not identified on baseline [^68^ Ga]Ga-HA-DOTATATE PET/CT, however only in 1 patient (1%) was this deemed a ‘true’ new lesion. This patient presented with new lesions after the second cycle of PRRT; multiple new bone metastases were identified on both planar imaging and SPECT/CT and is shown in Fig. 1. In the other 2 patients the lesions were not classified as new lesions during therapy as they were identified on the first post-therapy scan and therefore must have arisen during the time between the baseline PET/CT and first PRRT cycle. No additional new lesions were detected after subsequent cycles in these patients.

### Scan range

Sixteen patients (16%) had known bone metastases prior to start of PRRT that were visible only on planar imaging but out of range of the SPECT/CT. In 14/16 patients (88%) these metastases were located in the femur and/or cranium and this was deemed not clinically relevant due to the large number of bone metastases visible elsewhere and within the field of view of the SPECT/CT. The remaining 2/16 patients (12%) had a solitary bone metastasis (orbita, femur). However, this was also judged not clinically relevant with respect to response monitoring due to multiple evaluable soft tissue metastases within the range of the SPECT/CT.

Forty-five patients (45%) had disease visible only on the abdominal SPECT/CT, whilst 55 patients (55%) had disease visible on both abdominal and thoracic SPECT/CT. No patients presented with thoracic disease only. In 44/45 patients (98%) with abdominal disease only on baseline PET/CT, the thoracic SPECT/CT did not show any new lesions or other relevant additional findings during therapy. One patient did show new bone metastases after the second cycle within the thoracic skeleton. However, multiple new bone lesions were also elsewhere in the skeleton and clearly visible on abdominal SPECT/CT. Therefore, in this patient the thoracic SPECT/CT had no additional clinical value considering the assessment of disease progression during PRRT course.

### Additional clinical findings on low dose CT

In 11 patients (11%) a total of 14 additional findings were observed on the corresponding low dose non-contrast-enhanced CT scan. Three patients had two findings each. Some findings were seen during multiple cycles in the same patient, resulting in 25/368 scans with an additional finding (7%).

One patient presented with a non-specific lung nodule (1–2 mm) on the CT images after the first cycle of PRRT, which had no clinical consequences. Three patients showed new signs of swelling or enlargement of the intestines and presented clinically with bowel obstruction / ischemia after either the third or fourth cycle of PRRT. Pleural effusion was seen in four patients. The most frequent additional finding was ascites, which was present in 6 patients. A summary of the patients with additional findings, their symptoms and the interventions is given in Table 1. Three patients will be briefly discussed as illustration.

**Table 1:** Additional findings with corresponding symptoms and interventions

#	Clinical signs prior to PRRT	Additional finding	Change in clinical signs at time of additional finding	Intervention
8	Nausea, poor appetite, irregular defecation	C1-C4: ascites	Unchanged	None
9	Daily flushing, tube feeding	C3-C4: ascites	C4: poor condition, palpitations	None, referral cardiologist
15	Daily diarrhea due to ileotranversostomia	C2: lung nodule C3-C4: pleural effusion	Unchanged	None
19	Daily flushing, abdominal pain	C2: ascites	Unchanged	Treated for ileus 2 weeks later
34	Daily diarrhea	C3-C4: pleural effusion	Unchanged	C4: exculpatory puncture (> 400 mL)
35	Frequent flushes, diarrhea, WHO status 3	C3: ascites, bowel ischemia	C3: abdominal pain, nausea/vomiting	C3: Treated for ischemia 2 weeks later based on progressive pain and vomiting
56	Progressive abdominal pain, obstipation/diarrhea	C3-C4: bowel widening	C4: edema of the ankles	None
69	Diarrhea	C1-C4: pleural effusion	C3: lack of energy	C1 and C4: pleural puncture and drainage
87	Abdominal pain, tube feeding, diarrhea	C1-C4: ascites C2-C4: pleural effusion	Unchanged	None
94	Daily flushes	C3: ileus	C3: loss of apetite	None
98	No symptoms	C3: ascites	Unchanged	None

C1 = cycle 1, C2 = cycle 2, C3 = cycle 3, C4 = cycle 4

**Fig. 2:**
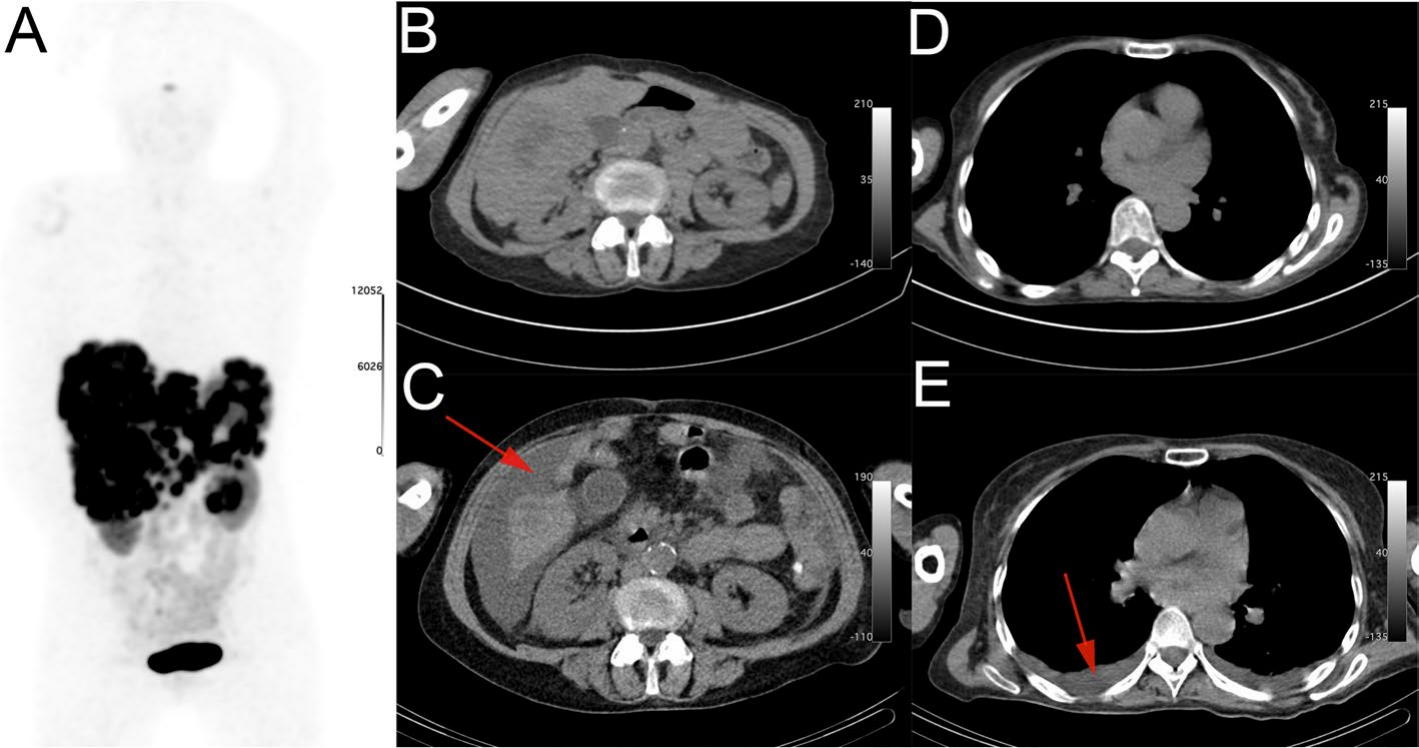
Maximum intensity projection of pre-therapy [^68^ Ga]Ga-HA-DOTATATE PET/CT (**A**). Pre-therapy CT (**B**) shows no ascites, which does appear on post-therapy CT after cycle 4 (arrow) (**C**). Pre-therapy CT (**D**) without pleural effusion, which appears on post-therapy CT after cycle 4 (**E**)

Patient #9 started PRRT for a grade 2 NET of unknown origin with extensive liver metastases, shown in Fig. 2. After the third cycle a trace of ascites was seen (asymptomatic), which progressed significantly on the post-PRRT imaging in the fourth cycle, which also showed pleural effusion. At that time a new progressive heart murmur was heard on physical examination and she was diagnosed with right sided heart failure and serious tricuspid valve regurgitation. She had an electrocardiogram and was seen by a cardiologist. However, she died of heart failure 2 weeks after the fourth cycle of PRRT.

**Fig. 3:**
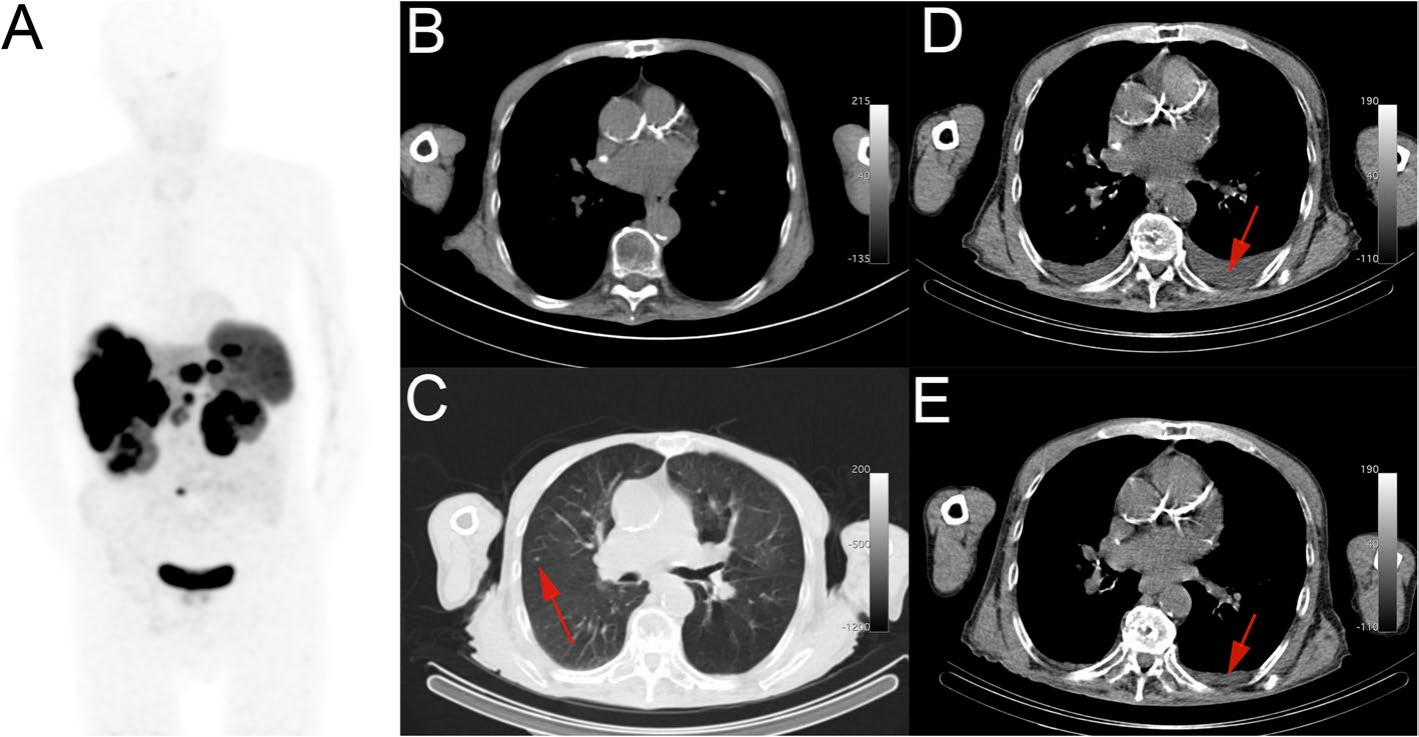
Maximum intensity projection of pre-therapy [^68^ Ga]Ga-HA-DOTATATE PET/CT (**A**). Pre-therapy CT (**B**), post-therapy CT image after cycle 2 shows a non-specific new lung lesion (arrow) (**C**). After cycle 3 pleural effusion appeared (**D**), which again decreased after cycle 4 (**E**)

Patient #15 underwent PRRT for a grade 1 pancreatic NET with lymph node-, liver-, and bone metastases. On the CT images after the second PRRT cycle, a nonspecific new lung nodule was found, which remained without clinical consequences as it was too small to diagnose or treat. After the third PRRT cycle asymptomatic pleural effusion was visible in both lungs on post-PRRT SPECT/CT (Fig. 3), which spontaneously decreased as observed on the fourth post-PRRT imaging and subsequently disappeared on follow-up scans. Nine months after the last PRRT, pleural effusion again arose and the patient died due to disease progression and bowel obstruction 4 months later.

**Fig. 4:**
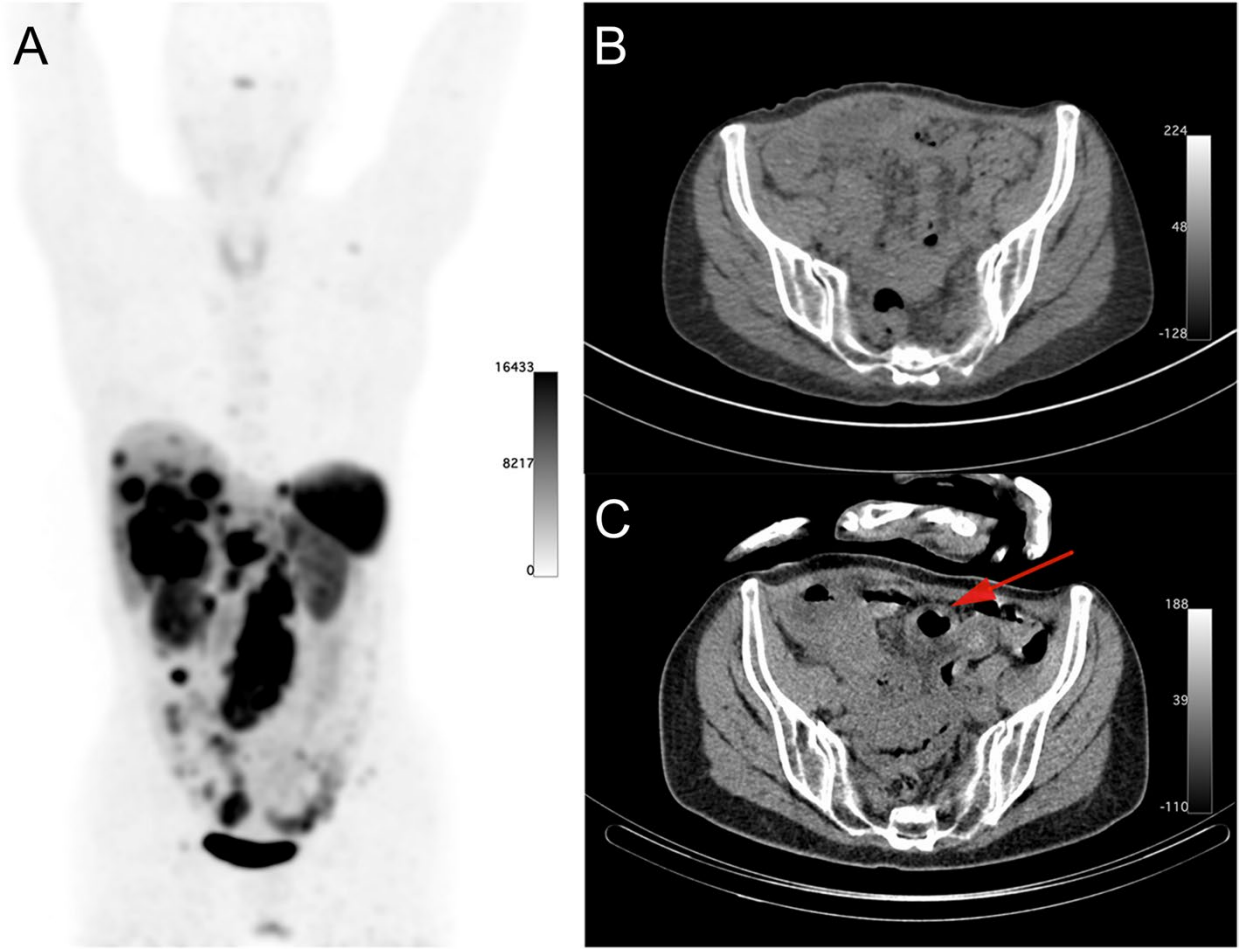
Maximum intensity projection of pre-therapy [^68^ Ga]Ga-HA-DOTATATE PET/CT (**A**). The pre-therapy CT showed no abnormalities (**B**), but after the third PRRT cycle swelling of the intestinal wall can be seen on the CT scan (**C**)

Patient #35 received PRRT for a grade 1 NET of the ileum with lymph node-, liver-, mesenteric-, and bone metastases. Three days prior the third cycle of PRRT she suffered from new abdominal pain and vomiting. The PRRT was administered as planned and post-therapy SPECT/CT imaging showed swelling of the intestine in the right abdomen (venous compression by mesenteric tumors), shown in Fig. 4, and air in the pelvis. She was diagnosed with intestinal ischemia. A nasogastric tube was placed and pain medication was given, which resulted in clinical improvement. The fourth PRRT cycle could be administered as planned ten weeks later. However, the patient passed away 6 months later after multiple episodes of bowel obstructions.

### Post-hoc analysis of ascites and overall survival

Since patients who developed ascites during the course of PRRT, seemed to have a short survival, a post-hoc analysis was performed to investigate the effect of ascites on overall survival (OS) from start of PRRT. For all patients the follow-up time was calculated from the start of PRRT to death of any cause or censored at last follow-up. Patients were divided into two groups: (1) no ascites on any post-therapy SPECT/CT scans, (2) ascites visible at any point on post-therapy SPECT/CT scans. A Kaplan–Meier curve was generated, and mean OS and the 95% confidence interval was estimated (since median OS was not reached in both groups), and a log-rank test was performed to determine whether OS was different between the two groups.

**Fig. 5:**
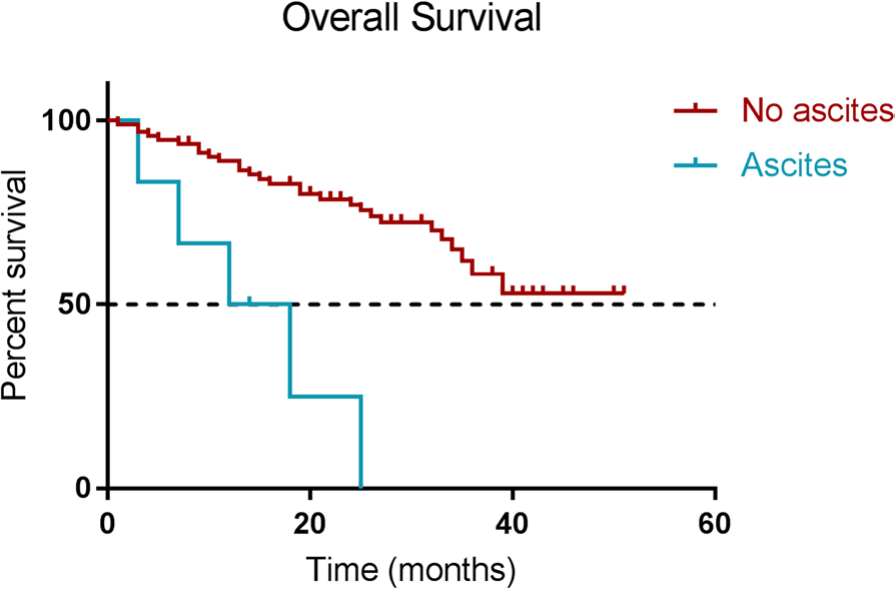
Kaplan–Meier curve for overall survival from start of PRRT for patients who developed ascites during the course of PRRT (blue) and patients that did not develop ascites (red)

Six patients (6%) showed ascites at any point during PRRT on post-therapy scans and there characteristics are shown in Table 2. Median follow-up time was 9.5 months [IQR 7–14], with a mean OS of 13.2 months (95% CI: 5.3–21.0 months). The remaining 94 patients (94%) did not develop ascites. Median follow-up time was 26 months [IQR 14–34] with a mean OS of 37.9 months (95% CI: 34.1–41.8 months). OS was significantly different between the two groups (*p* < 0.001) and the Kaplan–Meier curve is shown in Fig. 5.

**Table 2:** characteristics of patients with ascites

Case	Grade	Primary tumor	Tumor locations
8	2	Small intestine	Liver, LN, mesenteric
9	2	Unknown	Liver
19	1	Small intestine	Liver, LN, bone, lung, mesenteric, peritoneal
35	1	Small intestine	Liver, LN, bone, mesenteric
87	1	Caecum and rectum	LN, bone, lung, mesenteric, peritoneal
98	3	Pancreas	Liver, LN, pancreas

*LN* Lymph node

## Discussion

Post-PRRT gamma scintigraphy with either whole-body planar imaging and/or SPECT/CT has become standard of care in most centers. The discussion on the relevance, timing, number, and type of scans that are required however, is fueled predominantly by the discussion about dosimetry. To our knowledge, this is the first study to examine the value of post-PRRT imaging and the clinical relevance of the imaging protocol. Based on the absence of clinically relevant tumor lesions (i.e. relevant for clinical management during PRRT course) outside the field of view of the SPECT/CT and the presence of additional findings on low-dose CT, we suggest to use SPECT/CT over planar imaging in this patient population. The cohort included represents the patients at our hospital referred for PRRT, which are mainly G1/G2 GEP-NET. These results should be interpreted as such.

### Scan protocol

Whole-body planar imaging (15–20 min) combined with hemi-body (2 bed positions) SPECT/CT (40 min) leads to a total acquisition time of approximately 75 min. This is a high burden for both the patient and the nuclear medicine department. To assess the possibilities for protocol simplification and scanning time reduction, three factors were taken in to account: possibility of identifying new lesions, evaluation of suitable scan range compared to disease localization, and observation of additional findings.

In general, the sensitivity for lesion detection on SPECT is higher than on planar imaging, especially for smaller lesions. Furthermore, not all lesions are seen on planar imaging due to superimposition of other tumor lesions or organs with a high uptake. It can be argued that for the detection of new lesions in the head/neck region and extremities, whole-body planar imaging is of additional clinical value to (thoraco)abdominal SPECT/CT. However, the chance of detecting new metastases during PRRT course as such yet appeared very low; in the 100 included patients in this study (with 368 post-therapy imaging evaluations), only 1 patient developed new lesions during treatment which was visible on both planar and SPECT imaging. If clinically relevant disease progression that demands discontinuation of PRRT occurs, it is less likely that this arises in the extremities or head/neck region only, and will be missed on thoracoabdominal SPECT/CT. Therefore, for the purpose of new lesion detection, whole-body planar imaging seems of very low additional value to SPECT/CT. In 98% of patients that presented with only abdominal disease at baseline, no metastases were found outside of the abdomen during PRRT. In one patient the disease spread outside of the abdominal area, however progression (new metastases) was seen within the abdominal area as well. Therefore, the thoracic SPECT/CT appeared of very low additional value in patients with abdominal disease only, saving 20 min of imaging time.

### Ascites

The most common additional finding on low dose CT imaging was the appearance of ascites in 6 patients. Ascites often is an indication of end-stage cancer in general, and neuroendocrine tumors seem to be no different. Survival, depending on cancer type, typically lies around 6 months but can range up to 24 months. It is often caused by obstructed lymphatic drainage, increased vascular permeability in the tumor and/or liver disease/failure. Ascites can present with, sometimes unclear, symptoms of nausea, appetite loss, abdominal distension and pain and often detrimentally affect quality of life [8]. The mean OS of the patients in this study that developed ascites during PRRT was 13 months. This is considerably shorter than the median OS after PRRT which has been reported to be up to 82 months [9]. Answering the question whether patients that develop ascites during PRRT should continue treatment, or whether clinical management should shift to palliative supportive care focused on quality of life is beyond the scope of this study. Nevertheless, our observation underlines the importance of critical assessment of the low-dose CT images in post-therapy SPECT/CT imaging which can be of clinical value in interpreting symptoms reported by the patients during PRRT. Planar imaging did not reveal any relevant additional findings and for this purpose can be omitted when SPECT/CT is performed.

### Limitations

The retrospective comparison between whole-body planar imaging and hemi-body SPECT/CT imaging is not only a comparison of field of view but also of differences in technique. SPECT/CT has a higher resolution and sensitivity, whereas superimposition of tissues and/or lesions is a disadvantage of planar gamma imaging. However, we believe that whole-body SPECT/CT is currently unattainable due to the long acquisition time required with conventional SPECT/CT systems, although new generation scanners could provide whole-body SPECT/CT within an acceptable time limit. Furthermore, SPECT/CT always implies additional radiation dose to the patient. However, the extra radiation dose is low (2–5 mSv) with respect to the 370 mSv that the patient received by a single PRRT cycle [10]. Therefore selecting the most sensitive modality (SPECT/CT) for a limited but well-chosen range provides in essence all clinically relevant imaging information without putting a high burden on the patient and imaging department logistics.

### Recommendations

This study has resulted in several recommendations and changes in clinical practice in our institute. Firstly, planar imaging becomes redundant if SPECT/CT scans are acquired after PRRT. Secondly, in patients with abdominal disease only a post-therapy abdominal SPECT/CT is regarded sufficient and a thoracic SPECT/CT is not necessary but can be performed on indication. Furthermore, careful review of the CT images for additional findings, especially the development of ascites, is paramount. Lastly, SPECT/CT is now the preferred method of choice for dosimetry as well [11].

## Conclusion

From a clinical standpoint, SPECT/CT imaging is the preferred method for post-PRRT imaging and should be chosen over planar imaging. In patients with baseline abdominal disease only, SPECT/CT of the abdomen seems sufficient; in all other cases hemi-body (thoracic and abdominal) SPECT/CT should be performed. All accompanying low-dose CT images should be critically reviewed for additional findings, especially ascites, which is suggested to be a poor prognostic factor in patients receiving PRRT.

## Data Availability

The datasets used and/or analysed during the current study are available from the corresponding author on reasonable request.

## Abbreviations

CT: Computed tomography
HA-DOTATATE: High affinity DOTATATE, DOTA-3-iodo-Tyr3-octreotate
PRRT: Peptide receptor radionuclide therapy
SPECT: Single photon emission computed tomography
